# Discoidin Domain Receptor 2 Expression as Worse Prognostic Marker in Invasive Breast Cancer

**DOI:** 10.1155/2022/5169405

**Published:** 2022-03-07

**Authors:** Irene Romayor, Marina Luque García-Vaquero, Joana Márquez, Beatriz Arteta, Ramón Barceló, Aitor Benedicto

**Affiliations:** ^1^Department of Cell Biology and Histology, School of Medicine and Nursing, University of the Basque Country (UPV/EHU), 48940 Leioa, Spain; ^2^Department of Medicine and Cytometry General Service-Nucleus, CIBERONC, Cancer Research Centre (IBMCC/CSIC/USAL/IBSAL), 37007 Salamanca9, Spain; ^3^Oncology Service, Basurto University Hospital, 48002 Bilbao, Spain

## Abstract

Discoidin domain receptor 2 (*DDR2*) is arising as a promising therapeutic target in breast carcinoma (BC). The ability of *DDR2* to bind to collagen promotes protumoral responses in cancer cells that influence the tumor microenvironment (TME). Nonetheless, the interrelation between *DDR2* expression and TME modulation during BC progression remains poorly known. For this reason, we aim to evaluate the correlation between intratumoral expression of *DDR2* and the infiltration of the main TME cell populations, cancer-associated fibroblasts (CAFs), and tumor-associated macrophages (TAMs). First, collagen and *DDR2* expression levels were analyzed in human invasive BC samples. Then, *DDR2* status correlation with tumor aggressiveness and patient survival were retrieved from different databases. Subsequently, the main pathways, cell types, and tissues correlated with *DDR2* expression in BC were obtained through bioinformatics approach. Finally, we studied the association of *DDR2* expression with the recruitment of CAFs and TAMs. Our findings showed that, together with the expected overexpression of TME markers, *DDR2* was upregulated in tumor samples. Besides, we uncovered that altered TME markers were linked to *DDR2* expression in invasive BC patients. Consequently, *DDR2* modulates the stromal reaction through CAFs and TAMs infiltration and could be used as a potential worse prognostic factor in the treatment response of invasive BC.

## 1. Introduction

Breast carcinoma (BC) is the most prevalent malignancy in women and the leading cause of death among female population [1, 2]. Approximately 80% of all BC cases diagnosed correspond to invasive ductal carcinoma (IDC). Invasive lobular carcinoma (ILC) represents the second most common subclass of BC, with an incidence of around 15% [3]. Both subtypes differ in clinical, pathological, and biological aspects, with the expression of E-cadherin being the main hallmark of IDC [4]. Apart from its histology, BC tumor is classified according to its molecular phenotype regarding the presence of three different biomarkers: estrogen receptor (*ER*), progesterone receptor (*PR*), and human epidermal growth factor receptor 2 (*HER2*). Thus, luminal-like BC is characterized by *ER/PR* positive and *HER2* negative, *HER2*-enriched is defined by *ER/PR* negative or positive and *HER2* positive, and triple-negative tumor is described by the absence of these markers [5]. Current adjuvant treatments are based on targeting these proteins before or after surgery [6, 7], excepting triple-negative BC, for which chemotherapy in combination with immunotherapy regimens are commonly used [8]. However, despite medical advances, more than 20% of women with early-stage BC will suffer metastatic disease [9], which reflects the need for new targeted therapies.

One of the risk factors involved in BC malignancy is the dysregulation of extracellular matrix (ECM) composition and architecture, which is associated with the menopausal status [10] and predisposes women to develop invasive BC later on [11]. Aberrant ECM remodeling is characterized by a progressive collagen enrichment or desmoplasia, which increases mammary gland stiffness and alters the tissue mechanosignaling [12]. Collagen deposition and crosslink provide biochemical and biophysical signals to enhance diverse protumoral characteristics, regarding proliferation, migration, and invasion [13]. Several proteins, such as integrins and specific tyrosine kinase receptors (RTKs), mediate the crosstalk between cancer cells and ECM components [14]. One of the receptors involved in the tumor-ECM interplay is the discoidin domain receptor 2 (*DDR2*), an atypical RTK, due to its capacity to bind collagens [15].

*DDR2* overexpression has been linked to progression of different cancer types [16], including BC [17–19]. The role of *DDR2* in BC development has been uncovered recently, with special emphasis on its ability to stimulate the secretion of collagen-dependent proteases by tumor cells in postpartum-related carcinomas, activating their migratory and invasive potentials, as well as triggering metastasis [20]. Interestingly, apart from its implication on cancer cells response, *DDR2* receptor also mediates key BC cells' functional features that influence the phenotype and reaction of stromal cells, particularly cancer-associated fibroblasts (CAFs) [21, 22]. CAFs conform to the major component of the cellular fraction of the tumor microenvironment (TME) and actively participate in the desmoplastic process as major producers of extracellular collagen [23]. CAFs increase cancer cells growth through various mechanisms, including proliferation, migration, cytokine and chemokine secretion, and resistance to anticancer drugs [24, 25]. As well as CAFs, tumor-associated macrophages (TAMs) are a very relevant stromal population during ECM reorganization and carcinogenesis, due to the secretion of several cytokines and chemokines that create a favorable microenvironment for tumor growth, promoting cancer cells proliferation, migration, and chemoresistance [26]. Although CAFs and TAMs communication with tumor cells is essential for BC progression, the role of *DDR2* during this interaction remains unclear.

Given the importance of *DDR2* in invasive BC process, we evaluated collagen and *DDR2* status in tumor samples compared to healthy adjacent tissue, together with *DDR2* implication on metastasis progression. Besides, we studied the association of *DDR2* expression level and menopausal status with patient survival. Moreover, based on the capacity of *DDR2* to modulate the TME, we aimed to analyze the relation between *DDR2* expression and the recruitment of CAFs and TAMs into the breast tumors. Our findings revealed that collagen and *DDR2* expression levels were significantly higher in BC compared to normal adjacent tissue. In addition, *DDR2* upregulation was detected in metastatic tumors and represented a worse prognostic factor in combination with postmenopausal age. Moreover, *DDR2* overexpression correlated with a higher CAFs and TAMs infiltration into the mammary tumor tissue. Taking these results together, *DDR2* could play an important role in the modulation of the TME and may represent a potential target for antitumor early and metastatic BC treatments.

## 2. Materials and Methods

### 2.1. Patients and Tissue Samples

Breast IDC samples and the corresponding adjacent nontumoral or healthy tissues were obtained by mammary resections from 25 patients (*n* = 25). Adjacent nontumoral samples were collected from regions at least 5 mm away from tumor boundaries and were classified as normal breast tissue by a histopathologist. Although histological normalcy was detected, we must consider that carcinogenic alterations could affect the phenotypic and genetic profiles of the tissue regions up to 1 cm from the tumor margins [27]. Additionally, clinical and pathological features including menopausal status, tumor size, histological grade, tumor stage, proliferative index, e-cadherin expression, hormonal receptors-*HER2*/neu status, and lymph node invasion were recorded from the medical history (Table 1). Tumor staging was established according to the International Union against Cancer Tumour-Node-Metastasis (UICC-TNM) classification. All samples were provided by Basurto University Hospital Biobank (Basque Country, Spain) under the guidelines of the Institutional Ethics Committee with the corresponding informed consent.

### 2.2. Masson's Trichrome Staining

The detection of collagen type I fibers was performed through Masson's trichrome staining. In brief, 5 *μ*m tissue slides were incubated with Weigert's iron hematoxylin solution for 10 minutes and Biebrich scarlet-acid fuchsin Solution for 15 minutes. Finally, samples were treated with phosphomolybdic acid solution for 15 minutes and aniline blue solution for 5 minutes. All staining solutions were purchased from Sigma-Aldrich, United States (cat. numbers HT1079, HT151, HT153, and B8563, respectively). Collagen fibers were observed in blue, and expression level was quantified using FIJI-ImageJ (Colour Deconvolution plugin).

### 2.3. Immunohistochemistry and Scoring

The expressions of DDR2, *α*-SMA, and CD68 proteins were analyzed in consecutive sections of breast IDC and nontumoral samples. 5 *μ*m tissue slides were pretreated with Antigen Retrieval Solution (R&D Systems, United States, cat. number CTS013) for 10 minutes at 90°C. Endogenous peroxidase activity was blocked using 3% hydrogen peroxide (PanReac AppliChem, United States, cat. number 131077) in phosphate-buffered saline (PBS) 1X for 30 minutes at room temperature. Then, slides were incubated with 0.4% Triton X-100 (Thermo Fisher Scientific, United States, cat. number 85112) and 5% fetal bovine serum (FBS, Thermo Fisher Scientific, cat. number 10091148) in PBS 1X for 1 hour at room temperature and overnight at 4°C with one of the following primary antibodies: anti-DDR2 1 : 500 (GeneTex, United States, cat. number GTX25520), anti-*α*-SMA 1 : 200 (Dako, United States, cat. number GA61161-2), and anti-CD68 1 : 250 (Thermo Fisher Scientific, cat. number MA5-13324). Antibodies were detected with either biotinylated anti-rabbit 1 : 1500 or anti-mouse 1 : 2000 for 1 hour at room temperature, followed by streptavidin-HRP 1 : 500 for 30 minutes at room temperature and DAB Substrate Kit for 2-3 minutes (all purchased from Thermo Fisher Scientific, cat. numbers 31820, 31800, SA10001, and 34002, respectively). Finally, slides were counterstained in hematoxylin and mounted with Sub-X Mounting Medium (Leica Biosystems, Germany, product ID SUB-X-MOUNTING-MEDIUM). Protein expression was quantified using FIJI-ImageJ (Colour Deconvolution plugin).

The expressions of DDR2, *α*-SMA, and CD68 proteins were evaluated in the whole tissue for intensity of staining comparing breast IDC samples to nontumor ones. Staining with relative values up to 3-fold increase was classified as low status, while that with values superior to 3-fold increase was classified as high status. This evaluation was made following the criteria described by Toy et al. [28]. Samples 4, 12, and 23 (*α-SMA* staining), as well as 23 and 24 (*CD68* staining), were excluded from the correlation analysis due to the high percentage of fatty tissue detected, which made it complicated to classify them as low or high status.

### 2.4. RT-qPCR

Total RNA was extracted and purificated from breast IDC and nontumoral samples (including the stromal compartment) using the Total RNA Purification Kit (Norgen Biotek Corp., Canada, cat. number 17250). RNA concentration and quality were assessed by NanoDrop spectrophotometer (ND-1000; Thermo Fisher Scientific) and 0.5–1 *μ*g of RNA was reverse-transcribed into cDNA with iScript™ Reverse Transcription Supermix (BIO-RAD, United States, cat. number 1708841). Quantification of cDNA template was performed with real-time PCR using iTaq™ Universal SYBR® Green Supermix (BIO-RAD, cat. number 1725121) in ABI 7900HT (Life Technologies). PCR primers (Life Technologies) were as follows: *DDR2* F, GGAGGTCATGGCATCGAGTT, and R, GAGTGCCATCCCGACTGTAATT; *GAPDH* (housekeeping) F, GTATGACTCCACTCACGGCAA, and R, CTTCCCATTCTCGGCCTTG; *RPS15* (housekeeping) F, AGACGAGTTTCAGTGTTGCC, and R, AGACCACAGCCTCAGACAAG. Relative expression of target genes was normalized to the internal control genes *GAPDH* and *RPS15* by the ΔΔCt method. Data were generated by the use of specific software (ABI Prism, SDS2.0, Life Technologies) after normalization. The experiments were performed in triplicate.

### 2.5. Bioinformatic Analysis

For analysis of gene and *DDR2* expression correlation with TME markers, normal (*n* = 15) and invasive BC, IDC (*n* = 1237), and ILC (*n* = 116), microarray profiles generated by METABRIC project [29, 30] were retrieved from cBioPortal [31, 32]. Besides, *DDR2* expression profile based on the Cyclin D1 (*CCND1*) gene status as metastatic BC marker [33, 34] (with *CCND1* amplification, *n* = 12; without *CCND1* amplification, *n* = 80) and the effect of *DDR2* expression level and menopause status on patients survival (high *DDR2*/perimenopause, *n* = 9; high *DDR2*/postmenopause, *n* = 156; high *DDR2*/premenopause, *n* = 75; low *DDR2*/perimenopause, *n* = 28; low *DDR2*/postmenopause, *n* = 532; low *DDR2*/premenopause, *n* = 152) were obtained from UALCAN Cancer Database (datasets ID: MET500 and The Cancer Genome Atlas, TCGA) [35]. Then, the 100 most coexpressed genes with *DDR2* in invasive BC (Table S1) were acquired from Gene Expression Profiling Interactive Analysis Database 2 (GEPIA2; datasets ID: The Cancer Genome Atlas Program, TCGA, and Genotype-Tissue Expression Project, GTEx) [36]. Finally, KEGG 2019 Human and ARCHS4 Tissues platforms were applied to explore the biological functions and tissue correlation of *DDR2* during BC by using the Enrichr Database [37].

### 2.6. Statistical Analysis

Data were analyzed using GraphPad Prism software (version 6) and are presented as the mean ± standard deviation (SD). Differences in gene and protein expression levels were analyzed by two-tailed Student's *t*-test. The association between *DDR2* and stromal markers expression was analyzed by Chi-square, Fisher's exact test, and Pearson's correlation coefficient. The criterion for significance was *p* ≤ 0.05 for all comparisons.

## 3. Results

### 3.1. Collagen and DDR2 Expression Are Upregulated in Human Invasive BC Compared to Nontumoral Tissue

**Figure 1:**
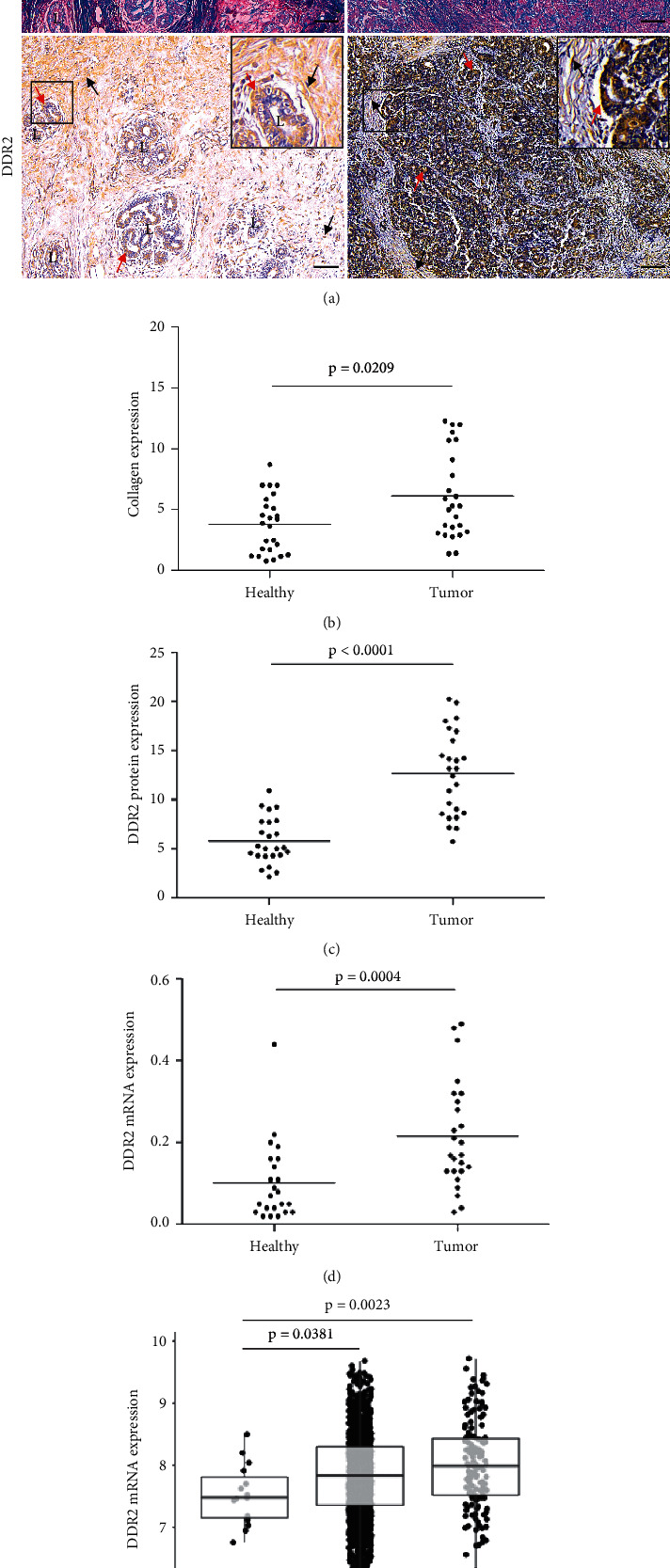
Expression of collagen and *DDR2* in human invasive BC. Masson's trichrome staining shows noncollagenous fibers in red (black arrow heads) and collagen type I fibers in blue (black arrows). Combination of both fibers appears in healthy breast tissue, while tumor tissue predominantly exhibits collagen expression. Immunohistochemistry shows that healthy mammary gland epithelial cells and tumor cells express *DDR2* (red arrows). Besides, *DDR2* staining is observed in the stroma of normal and tumor samples (black arrows). L: lobule, T: tumor, S: stroma. Scale bar: 100 *μ*m. Insets: 2x magnification (a). Staining quantification (colored area per total tissue area) indicates that collagen (*p*=0.0209) and *DDR2* (*p* < 0.0001) expressions are significantly higher in breast IDC samples (b, c). RT-qPCR demonstrates that relative expression of *DDR2* is significantly upregulated in breast cancerous tissues when compared to that in nontumoral tissues (including the stromal compartment) (*p*=0.0004) (d). Mean mRNA levels of *DDR2* are overexpressed in breast IDC (*p*=0.038) and ILC (*p*=0.0023) samples obtained from METABRIC BC Datasets (e). Data are expressed as the mean ± SD.

Collagen accumulation and *DDR2* high expression have been reported in invasive BC both *in vitro* and *in vivo* [17–19, 38, 39]. We analyzed the intratumoral levels of collagen type I and *DDR2* in 25 mammary tissue samples from IDC (e-cadherin positive) and their matched adjacent normal tissues. As observed in Figure 1, the vast majority of the tumors showed collagen deposition (*p*=0.0209) and upregulated *DDR2* protein (*p* < 0.0001) compared to that in nontumoral tissue. Besides, *DDR2* quantification in the RNA samples obtained from IDC patients supported this finding, as demonstrated by RT-qPCR (*p*=0.0004). Further analysis of *DDR2* status using METABRIC Database confirmed that *DDR2* mRNA levels were significantly higher in ductal and lobular BC subtypes, with respect to those in the normal breast tissue (*p*=0.0381 and *p*=0.0023, respectively).

### 3.2. DDR2 Upregulation Is Associated with BC Metastatic Progression and Low Postmenopausal Patient Survival

**Figure 2:**
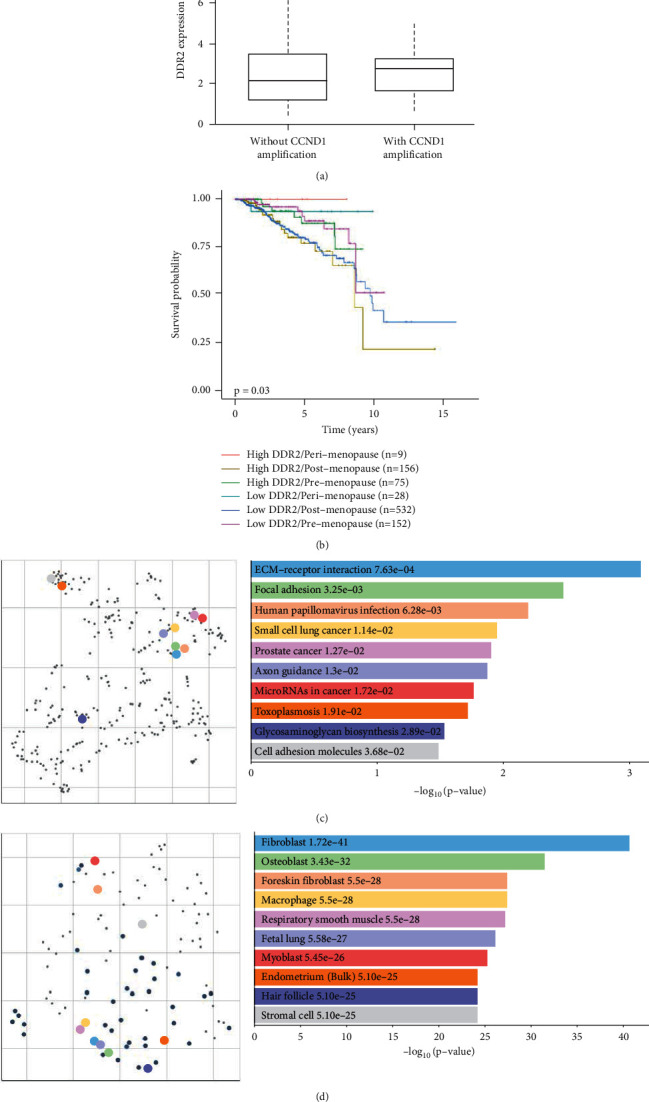
Implication of *DDR2* expression on invasive BC progression, patients' outcome, and cell signaling pathways. UALCAN Cancer Database reveals that mean mRNA levels of *DDR2* are upregulated in breast tumor samples with *CCND1* gene amplification compared to those without *CCND1* overexpression (*p*=0.0318). RPKM: reads per kilobase of transcript (a). Moreover, concerning menopausal age (pre-, peri-, or postmenopause women), high *DDR2* expression in combination with postmenopausal status significantly decreases patient survival compared to low *DDR2* status (*p*=0.03). The comparison between the other groups does not show significant results (*p* > 0.05) (b). KEGG 2019 Human pathways (c). and ARCHS4 Tissues expression (d). correlation with *DDR2* in invasive BC. Scatter plots (left) represent gene clusters according to their similarity on a map. Colored circles correspond to the Top 10 enriched terms visualized in the bar chart together with their *p* values (right).

*DDR2* overexpression has been related to poor prognosis in several cancer types, including BC [18, 28]. As observed in Figure 2, metadata analysis showed that *DDR2* gene level was higher in those invasive BC tumors with a more aggressive phenotype (*p*=0.0318), defined by the *CCND1* amplification as metastatic marker [33]. Moreover, regarding patient age, high *DDR2* expression notably increased postmenopausal BC patients' mortality, compared to the postmenopausal women who exhibited low *DDR2* expression levels (*p*=0.03). On the contrary, *DDR2* expression was not relevant in pre- and perimenopausal women. Thus, *DDR2* status was only significant when we analyzed postmenopausal BC patients.

### 3.3. DDR2 Expression in BC Is Corrlated with Cancer-Associated Genes and Stromal Cells Markers

In order to evaluate the role of *DDR2* during BC development, we studied the function of the main genes coexpressed with *DDR2* in the mammary tumor tissue. On the one hand, KEGG 2019 Human Database indicated that *DDR2* was predominantly related to ECM interplay and cell adhesion, two features directly linked with DDR tyrosine kinase family, along with cancer signaling pathways. On the other hand, ARCHS4 Tissues Database revealed that 4 out of 10 of the most associated cells with *DDR2* in BC were from fibroblast origin (fibroblasts, foreskin fibroblasts, and myoblasts), together with macrophages, thus showing a stromal pattern (Figure 2).

### 3.4. Enhanced Expression of *α*-SMA and CD68 in Human Breast IDC

**Figure 3:**
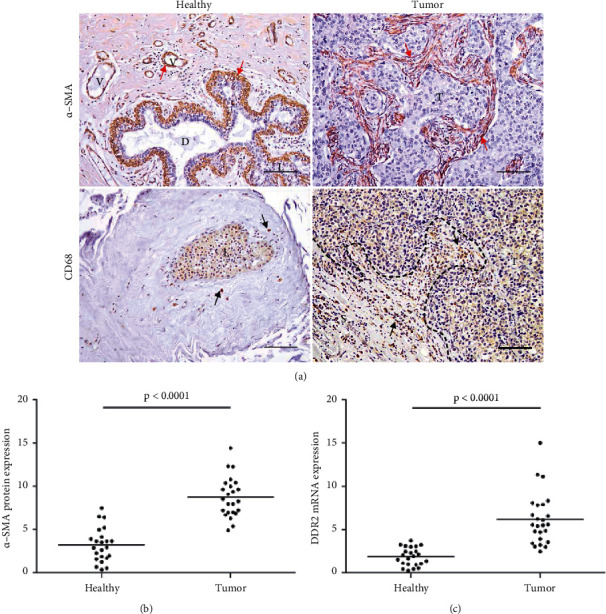
Expression of *α-SMA* and *CD68* in nontumoral breast tissue and breast IDC samples. Immunohistochemistry shows that normal myoepithelial cells and vessels (V) express *α-SMA*. Stromal fibroblasts from tumor samples also express *α-SMA* (red arrows). Stromal macrophages from normal and tumor tissues express *CD68* (black arrows). D: duct; L: lobule; T: tumor, S: stroma. Scale bar: 50 *μ*m (a). Staining quantification (colored area per total tissue area) shows that stromal cells markers expression is significantly upregulated in breast IDC samples (*p* < 0.0001) (b, c). Data are expressed as the mean ± SD.

CAFs and TAMs are active players in BC progression and chemoresistance [25, 26]. We evaluated the infiltration of CAFs and TAMs in breast IDC samples by *α-SMA* and *CD68* staining quantification, respectively. In the healthy breast tissue, these markers correspond to fibroblast-like cells and macrophages, which maintain mammary gland homeostasis. Thus, the comparison of *α-SMA* and *CD68* expression levels between normal and tumor samples represents the mobilization of these stromal cell populations during the tumorigenic process. As observed in Figure 3, we found increased expressions of *α-SMA* and *CD68* markers in the peritumoral and intratumoral areas compared to normal adjacent tissue. These results revealed a pronounced recruitment of CAFs and TAMs in the developing tumoral tissue from breast IDC patients.

### 3.5. Overexpression of DDR2 Correlates with Increased CAFs Infiltration in Human Invasive BC

**Figure 4:**
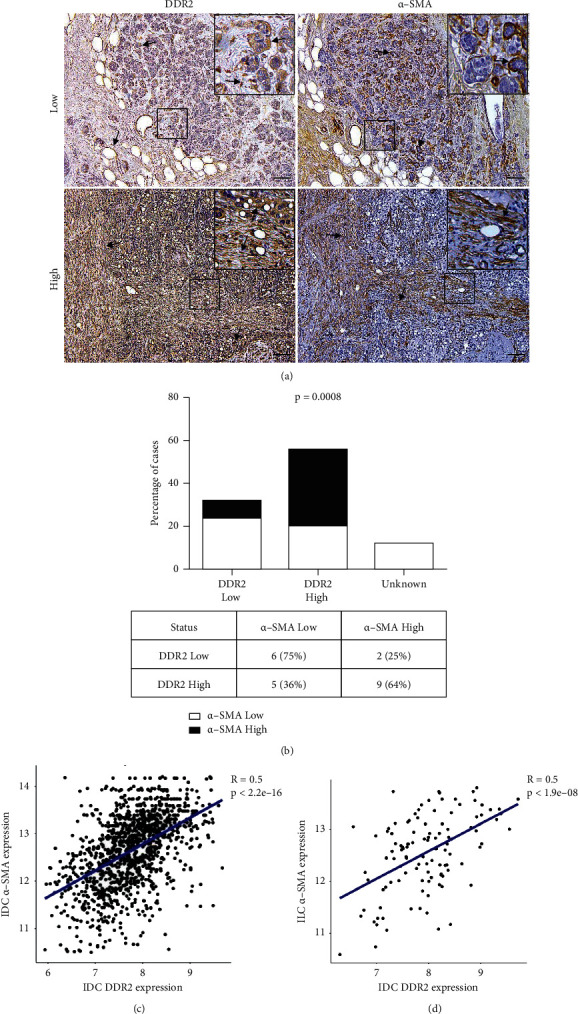
Interplay between the expressions of *DDR2* and *α-SMA* in human invasive BC. Images represent characteristic immunohistochemical samples of *DDR2* and *α-SMA* low and high expression (arrows). Scale bar: 100 *μ*m. Insets: 2x magnification (a). Graph shows that *DDR2* expression is related to *α-SMA* levels (*p*=0.0008) (b). *DDR2* and *α-SMA* status positive correlation in breast IDC (*R* = 0.5; *p* < 2.2*e* − 16) and ILC (*R* = 0.5; *p* < 1.9*e* − 08) samples obtained from METABRIC BC Datasets (c, d).

The TME is a dynamic compartment that drives tumor growth. CAFs are a major player in this ecosystem, supporting BC growth through several pathways [25]. We aimed to elucidate whether the intratumoral *DDR2* expression may be related to the recruitment of CAFs and, therefore, could be used as a marker of stromal reorganization during BC. Using Chi-square and Fisher's exact test, we found a strong correlation between intratumoral *DDR2* protein levels and CAFs infiltration (characterized by *α-SMA* expression) in breast IDC samples. As observed in Figure 4, low *DDR2* expression was directly linked with reduced CAFs recruitment (*p*=0.0008). In depth, 75% of the low *DDR2* expressing tumors exhibited low *α-SMA* staining, while CAFs infiltration was high in only 25% of the low *DDR2* levels breast tumors. On the other hand, 64% of the high *DDR2* expressing tumors showed elevated *α-SMA* protein levels, while barely 36% of these tumors presented low *α-SMA* expression. Additional analysis of *DDR2* and *α-SMA* correlation in IDC and ILC using METABRIC Database supported this finding (*p* < 2.2*e* − 16 and *p* < 1.9*e* − 08, respectively), uncovering a direct relation between tumoral *DDR2* expression and CAFs recruitment.

### 3.6. TAMs Recruitment Correlates with DDR2 Upregulation in Human Invasive BC

**Figure 5:**
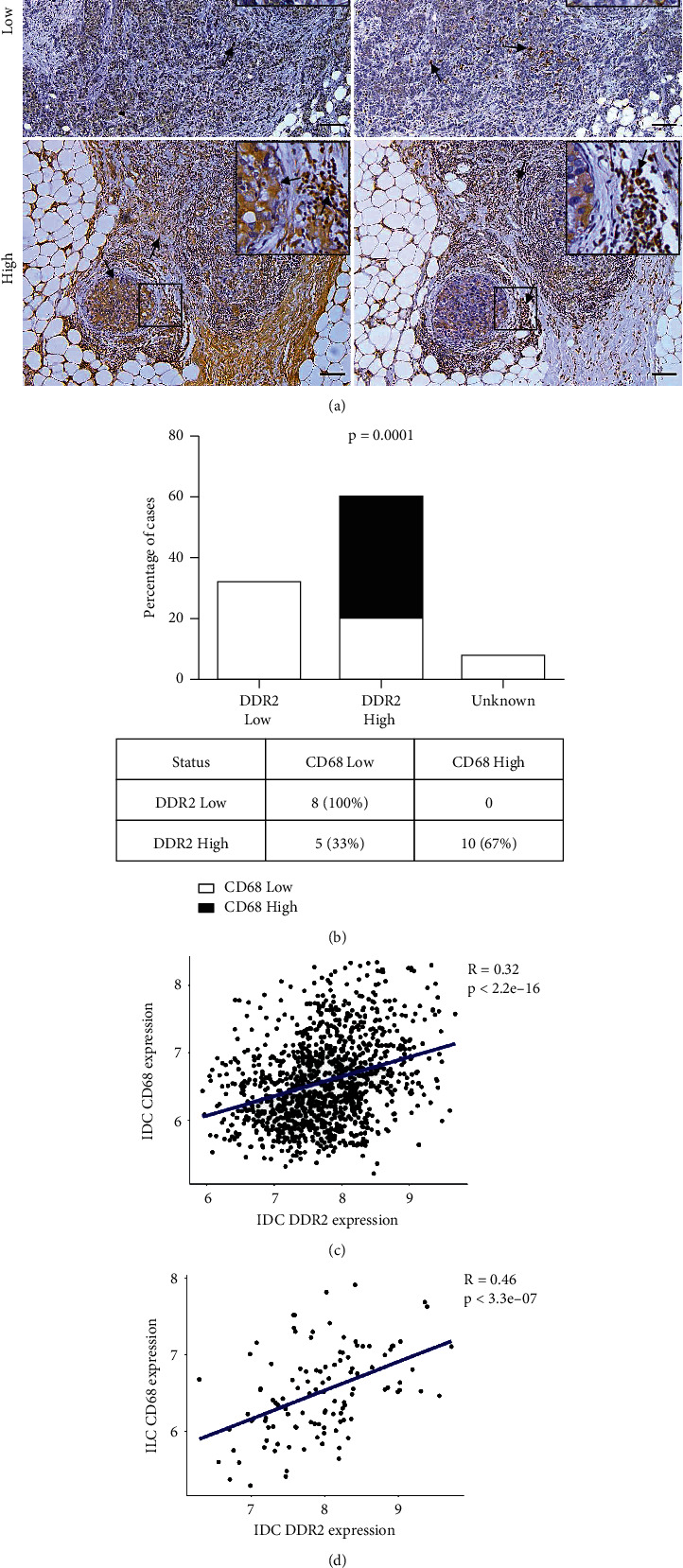
Interplay between the expressions of *DDR2* and *CD68* in human invasive BC. Images represent characteristic immunohistochemical samples of *DDR2* and *CD68* low and high expression (arrows). Scale bar: 100 *μ*m. Insets: 2x magnification (a). Graph shows that *DDR2* expression is related to *CD68* levels (*p*=0.0001) (b). *DDR2* and *CD68* status positive correlation in breast IDC (*R* = 0.32; *p* < 2.2*e* − 16) and ILC (*R* = 0.46; *p* < 3.3*e* − 07) samples obtained from METABRIC BC Datasets (c, d).

TAMs, key immunomodulatory cells, are a significant source of cytokines and chemokines that promote disease progression [26]. Here we analyzed the correlation of infiltrating *CD68* positive macrophages and the expression of intratumoral *DDR2* in breast IDC samples. Chi-square and Fisher's exact test revealed a deep correlation between high intratumoral *DDR2* expression and elevated infiltration of *CD68* positive TAMs (*p*=0.0001). Strikingly, low macrophages counts were found only in patients with low levels of intratumoral *DDR2*. However, 67% of high *DDR2* expressing tumors showed elevated macrophages recruitment, and only 33% of these tumors presented low *CD68* positive cells counts (Figure 5). Further analysis of *DDR2* and *CD68* correlation in IDC and ILC using METABRIC Database confirmed our results (*p* < 2.2*e* − 16 and *p* < 3.3*e* − 07, respectively), suggesting a feasible link between intratumoral *DDR2* expression and TAMs infiltration.

## 4. Discussion

The ECM is one of the main components of the TME. The importance of these extracellular elements resides in their ability to promote significant changes in the behavior of cancer cells through receptor-mediated ECM-tumor cell interactions. These alterations exert a positive effect on cancer cells, by means of chemoresistance, as well as increased proliferation and migration, among others [40]. Regarding BC, the first most prevalent malignant disease among women, collagen accumulation has been postulated as a risk factor [38, 41]. Despite the limited number of patients, and taking into account the fact that the heterogeneity of the BC population represents a limiting factor, the analyzed invasive BC tissue samples exhibit a marked collagen deposition, pointing out the crosstalk between ECM and BC cells as a feasible starting point for tumor development. Even though collagen constitutes an integral and functional key player of cancer tissue regulation, current therapies against the variety of histological and molecular BC subtypes do not target cellular components directly linked with this ECM protein.

The collagen-binding RTK *DDR2* is known to play important roles in tumor progression [16]. In the mammary carcinogenic tissue, *DDR2* expression has been postulated as an independent prognostic value [18], with a critical role during postpartum-associated BC [20, 42]. In this regard, increased *DDR2* promotes tumor aggressiveness in the breast, by means of enhanced proliferation, migration, colony formation, and metastasis [17, 19]. In this work, we show that *DDR2* expression is upregulated in a small sample size composed by different invasive BC molecular profiling cohorts, including luminal-like, *HER2*-enriched, and triple-negative subclasses. Besides, the later bioinformatics analysis including a large number of patients confirms these results. As for the last, Toy et al. [28] described that high *DDR2* protein levels were significantly associated with poor triple-negative BC patients' outcome, evidencing the involvement of *DDR2* in tumor malignancy. In this line, we detect *DDR2* overexpression in metastatic BC patients with *CCND1* gene amplification, a worse survival predictive biomarker in breast tumors [33, 34]. Furthermore, we uncover that mortality increase in those women with a high *DDR2* expression status along with postmenopausal age. Thus, elevated expression of *DDR2* could reflect a worse prognosis in those postmenopausal BC patients. Taken together, these results suggest that *DDR2* may participate in tumor cells growth and permanence. In fact, bioinformatics analyses corroborate that *DDR2* signaling in BC is widely connected to cancer-associated pathways, apart from those related to ECM interaction. Interestingly, the examination of the *DDR2* expression pattern indicates that this receptor represents an important part of the stromal compartment, mostly detected in fibroblasts and macrophages. This goes in line with the immunohistochemical analysis, in which we observe *DDR2* expression in the tumor stroma of BC tissue slides.

The TME is known to support cancer progression, influencing therapeutic response and clinical outcome. Thus, infiltrating CAFs and TAMs, the main TME cell populations, maintain cancer development [43]. Regarding stromal cells markers detection in 25 BC patients, *α-SMA* and *CD68* upregulation reveals a high recruitment of these cells into the mammary tumor tissue. Concerning *α-SMA* detection, it is also found in vascular muscular cells and pericytes, which account in high densities in tumor tissues [44]. Even though the heterogeneity of CAFs in the TME cannot be described using a single marker, *α-SMA* expression has been extensively analyzed as one of the main myofibroblast markers of the tumor stroma [45, 46]. Intriguingly, adding our small number of samples with the extensive datasets available, we observe a correlation between *DDR2* overexpression and increased levels of CAFs and TAMs in the malignant tissue. This finding suggests that the stromal fraction of BC may be modulated by tumor *DDR2*. Aside from cancer cell intrinsic effects, *DDR2* is also involved in several processes that alter the breast TME, facilitating disease progression. This could be controlled by the wide spectrum of *DDR2* mediated changes in the tumor stroma. In this line, Corsa et al. proposed that *DDR2* expressed by BC cells determines CAFs activation to enhance tumor invasion and metastasis [22]. Similarly, tumor *DDR2* regulates collagen signaling in CAFs during BC progression, as recently reported by Bayer et al. [21]. Comparably, we uncover a plausible positive interrelation between the levels of *DDR2* and the mobilization of CAFs and TAMs into the tumor foci, which points out to *DDR2* as a possible regulator of attracting signals for these cell populations. Concerning the latter, it is well established that *DDR2* is implicated in metalloproteinases (MMPs) secretion by tumor cells [47], resulting in ECM degradation and facilitating stromal cells infiltration and cancer cells migration and invasion [48]. Thus, MMPs production by BC cells through *DDR2* modulation could promote CAFs and TAMs recruitment. Eventually, although CAFs and TAMs are defined as cancer progression promotors, these stromal cells can also delay tumor growth, which depends on the subtype of CAFs and TAMs populations [49–52]. Considering its dual role, additional analysis evaluating CAFs and TAMs subpopulations markers may elucidate their specific phenotype.

Other *DDR2* stimulated proteins are *TGF-β* and parathyroid hormone-related protein (*PTHrP*), known to play a role in bone metastasis [53]. On the one hand, *TGF-β* contributes to CAFs infiltration and proliferation, which produce excessive ECM deposition, providing a scaffold for the entrance of immune cells, such as TAMs, and a substrate for cell migration [54, 55]. On the other hand, PTHrP induces the epithelial-to-mesenchymal transition (EMT), by which epithelial cells transform in fibroblasts in several tissues [56, 57, 58]. This process may account for breast epithelium, therefore consisting in a fibroblast source related to *DDR2*. Interestingly, in bone metastasis, *PTHrP* seems to stimulate the recruitment of macrophages into the tumor promoting the secretion of *CCL2* by osteoblasts [56]. It is tempting to hypothesize that a similar process may occur in breast tissue, driving to TAMs accumulation.

This work represents one of the few preliminary studies correlating *DDR2* expression with CAFs and TAMs recruitment during invasive BC progression. However, further ongoing studies will expand the relation between these markers during BC development. Our prior conclusions reflect that *DDR2* may constitute an alternative therapeutic target, especially for those individuals with invasive BC or with a high risk of relapse, due to the ineffectiveness of hormone and *HER-2* therapies, like in triple-negative subtype, as well as the development of chemoresistance. The observed potential connection between *DDR2* and the TME markers could put some light on the treatment options for these patients, focusing on the combination of *DDR2* inhibitors and stromal-directed drug regimens with the present clinical approaches.

## Figures and Tables

**Table 1:** Clinicopathologic characteristics of BC patients (*n* = 25).

Characteristics	*N* (%)
Menopausal status (median age = 63,8 years)	
Premenopause	6 (24)
Postmenopause	19 (76)
Tumor size (cm)	
≤2.5	13 (52)
>2.5	10 (40)
Unknown	2 (8)
Tumor grade	
I	0 (0)
II	10 (40)
III	15 (60)
T stage	
T0	1 (4)
T1	4 (16)
T2	20 (80)
N Stage	
N0	10 (40)
N1	9 (36)
N2	5 (20)
N3	1 (4)
M stage	
M0	25 (100)
Proliferative index (Ki-67 staining)	
Low (<20%)	3 (12)
High (≥20%)	22 (88)
E-cadherin	
Positive (IDC subtype)	25 (100)
Negative (ILC subtype)	0 (0)
Hormonal receptors-*HER2*/neu status	
*ER/PR* (+)-*HER2* (−) (luminal-like)	9 (36)
*ER/PR* (+)-*HER2* (+) (*HER2*-enriched)	10 (40)
*ER/PR* (−)-*HER2* (+) (*HER2*-enriched)	2 (8)
*ER/PR* (−)-*HER2* (−) (triple-negative)	4 (16)
Lymphovascular invasion	
Present	15 (60)
Absent	10 (40)

## Data Availability

The data used to support the findings of this study are available upon request from Dr. Beatriz Arteta through the email address beatriz.arteta@ehu.eus.

## Supplementary Materials

Supplementary Materials Table S1: genes positively correlated with DDR2 in invasive BC (TOP 100). PCC: Pearson Correlation Coefficient.
