# Supplementary material for: Discoidin Domain Receptor 2 Expression as Worse Prognostic Marker in Invasive Breast Cancer
Source: Breast J. 2022 Mar 7;2022:5169405. doi: 10.1155/2022/5169405 (PMC9187291; doi:10.1155/2022/5169405)
Supplement: Supplementary Materials — Table S1: genes positively correlated with DDR2 in invasive BC (TOP 100). PCC: Pearson Correlation Coefficient. [file 5169405.f1.docx]

**Table S1.** Genes positively correlated with DDR2 in invasive BC (TOP 100). PCC: Pearson Correlation Coefficient.

| Gene symbol | PCC |  | Gene symbol | PCC |
| --- | --- | --- | --- | --- |
| BICC1  FSTL1  HEG1  RECK  PEAK1  FERMT2  MAGI2-AS3  KIRREL  DSE  FAT4  MSRB3  FOXN3  CLMP  CALD1  ARHGAP31  TCF4  ANTXR2  TRAM2  ZEB2  QKI  AC000403.4  ZEB1  PKD2  LUZP1  TIMP2  KCTD12  TNS1  C14orf37  VSTM4  OLFML1  ARHGAP20  RBMS3  ALDH1L2  LATS2  LAMA4  ECM2  PRRX1  NRP1  SEPT11  GAS7  ZFHX4  EPB41L2  ZFPM2  MXRA5  HMCN1  LOX  DCN  SH3PXD2A  LRCH2  RBMS1 | 0.75  0.74  0.69  0.69  0.69  0.69  0.68  0.68  0.68  0.68  0.68  0.68  0.67  0.67  0.67  0.67  0.67  0.67  0.66  0.66  0.66  0.66  0.66  0.66  0.66  0.66  0.66  0.65  0.64  0.64  0.64  0.64  0.64  0.64  0.63  0.63  0.63  0.63  0.63  0.63  0.62  0.62  0.62  0.62  0.62  0.62  0.62  0.62  0.62  0.62 |  | RFTN2  DPYSL3  PLSCR4  ADAM12  AXL  CDH11  MEF2C  SLIT2  ELK3  ITGB1  RP11-815J21.4  CHSY3  FAM26E  NID1  SH3D19  PROS1  VCAN  SEC23A  RBMS2  COL6A3  FOXO1  LHFPL2  MDFIC  CNRIP1  GLG1  CCDC80  NEXN  MRVI1  GXYLT2  BNC2  FBN1  PCSK5  PDGFRB  TRPC1  SOCS5  MSC-AS1  NAP1L3  MBNL1  TMEM43  PCDH18  ARHGAP28  CLIC4  C17orf51  LAMA2  DAB2  VGLL3  RBFOX2  CTTNBP2NL  EVC  PTRF | 0.62  0.62  0.62  0.62  0.61  0.61  0.61  0.61  0.61  0.61  0.61  0.61  0.61  0.61  0.61  0.60  0.60  0.60  0.60  0.60  0.60  0.60  0.60  0.60  0.60  0.60  0.60  0.60  0.60  0.60  0.60  0.59  0.59  0.59  0.59  0.59  0.59  0.59  0.59  0.59  0.59  0.59  0.59  0.59  0.59  0.59  0.59  0.59  0.58  0.58 |
